# Correlation of Microstructure, Rheological and Morphological Characteristics of Synthesized Polypropylene (PP) Reactor Blends Using Homogeneous Binary Metallocene Catalyst

**DOI:** 10.3390/polym9030075

**Published:** 2017-02-24

**Authors:** Javid Vaezi, Mehdi Nekoomanesh, Hossein Ali Khonakdar, Seyed Hassan Jafari, Alireza Mojarrad

**Affiliations:** 1 Iran Polymer and Petrochemical Institute (IPPI), P.O. Box 14965/115 Tehran, Iran; jv.araz@gmail.com (J.V.); M.nekoomanesh@ippi.ac.ir (M.N.); 2 National Petrochemical Company, Petrochemical Research and Technology Company (NPC-RT), P.O. Box 1435884711 Tehran, Iran; 3 Leibniz Institute of Polymer Research, D-01067 Dresden, Germany; 4 School of Chemical Engineering, College of Engineering, University of Tehran, P.O. Box 11155-4563 Tehran, Iran; shjafari@ut.ac.ir; 5 Department of Chemical Engineering, Faculty of Engineering, Shiraz Branch, Islamic Azad University, P.O. Box 71993-3 Shiraz, Iran; spic@petro-plastic.com

**Keywords:** binary metallocene catalyst, bimodal polypropylene, rheology, morphology

## Abstract

A novel binary homogeneous catalyst system based on (I): *rac*-Me_2_Si(2-Me-4-PhIn)_2_ZrCl_2_ and (II): (2-PhIn)_2_ZrCl_2_ catalysts at various molar ratios was utilized for the synthesis of polypropylene (PP) reactor blends with bimodal molecular weight distribution (MWD). The results of gel permeation chromatography analyses revealed that the catalyst (I) was responsible for the production of *i*-PP with high molecular weight (MW) while the individual use of catalyst (II) led to the production of an elastomeric PP with relatively low MW. However, application of the binary catalyst system led to high MW bimodal MWD products being highly dependent on the catalysts’ molar ratios. Increasing the molar ratio of catalyst (II) to catalyst (I) resulted in a notable enhancement of the products’ complex viscosity due to the increased MW, a higher level of chains’ entanglements and formation of amorphous blocks along the polymer chains. All products exhibited a single relaxation that shifted towards longer times upon changing the catalysts’ molar ratios. Scanning electron microscopy results revealed that the fracture surface of the blends, synthesized by the binary catalyst system, became more heterogeneous in comparison with the products obtained by the individual use of the catalyst (I). The observed heterogeneity was found to increase by increasing the amount of catalyst (II). Such morphological change was further corroborated by the dynamic rheological data, indicating a promising correlation between the linear rheological results and the morphological features of the synthesized PP reactor blends.

## 1. Introduction

**Scheme 1:**
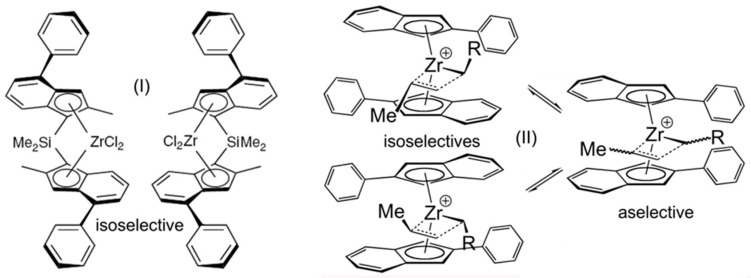
Precursor (I): *rac*-Me_2_Si(2-Me-4-Ph In)_2_ZrCl_2_ and (II): activated oscillating (2-Ph In)_2_ZrCl_2_ catalyst.

Polypropylene (PP) is one of the most commonly used plastics which is increasingly being utilized worldwide due to its rather low price, good processability and its ease of modification for different applications. Since being commercialized by Montedison Corporation in 1957 for the first time, a diverse range of catalysts and technologies have been developed for the fabrication of PP. However, the synthesis of PP with novel microstructure and tailored properties was further facilitated once the metallocene catalysts were developed [1,2,3,4,5,6,7]. Recent researches have been conducted with the aim of synthesizing polyolefins with improved microstructure and properties, and to this end, novel catalyst systems were designed. Of the new strategies to synthesize polyolefins with optimum properties, one could mention the alloy, hybrid and multi-component catalyst systems [8,9,10,11,12,13,14,15,16]. In those systems, each catalyst produces a polymer with a unique structure and properties. Due to the occurrence of chain transfer reactions from one active site to another, in the presence of a chain transfer agent such as trimethyl aluminum (TMA) or triisobutyl aluminum (TIBA), the final product is a reactor blend of two or more polymers possessing stereoblock copolymer microstructures, which exhibit different properties as compared to the polymers produced from the individual use of catalysts [17,18,19,20]. Several research groups successfully synthesized PP-based thermoplastic elastomers with excellent elastic properties, and also, stereoblock structures with the ability to compatibilize two heterogeneous polymer phases using a mixture of isospecific–aspecific and isospecific–syndiospecific metallocene catalysts [21,22]. Moreover, the synthesis of PP blends with novel properties possessing atactic, isotactic and stereoblock structures via the use of a mixture of methyl aluminoxane (MAO)-activated metallocene catalysts was reported [23]. Similar to other polymers, molecular weight (MW) and molecular weight distribution (MWD) are considered as crucial factors influencing the processing of PP. In spite of the good control over the MW of the synthesized products with tailored structure, which are regarded as the advantages of metallocene catalysts, the narrow MWD of the obtained polymers might limit the processability window and thus their potential applications. As is well-established, MW is responsible for the mechanical properties and MWD is highly influential on the processability and rheological properties of the final polymer. In many applications, such as blow molding, extrusion and packaging, polymers with bimodal MWD are needed to attain a product with high mechanical strength, stiffness and impact strength and also a good level of processability [24,25,26]. Since each of the catalysts within the binary catalyst systems is able to produce a unique polymer, it would be possible to achieve PPs with bimodal or broad MWDs if the catalyst system is properly designed. Therefore, some of the researchers used both homogeneous and heterogeneous binary metallocene silica-supported catalysts with different stereo-specificities to synthesize PPs with broad and bimodal MWD as well as stereoblock structures with different melting points [16,26,27,28,29,30,31,32,33,34]. Numerous studies have been conducted on the microstructural characterization of PPs synthesized by binary or hybrid catalyst systems as well as the effects of type and composition of catalysts on their microstructures through different characterization techniques [15,35,36,37]. However, there exists limited literature on the morphological and rheological properties of these systems and their relationship with the microstructure of the synthesized PPs. The aim of the current work is to study the microstructure, rheological and morphological characteristics of PP reactor blends with bimodal MWDs synthesized by a novel binary homogeneous system including (I): *rac*-Me_2_Si(2-Me-4-PhIn)_2_ZrCl_2_; and (II): (2-PhIn)_2_ZrCl_2_ catalysts (Scheme 1) at different molar ratios of the catalysts. In such binary systems, catalyst (I) is responsible for producing isotactic PP (*i*-PP) with high MW and catalyst (II) results in the production of an elastomeric PP with relatively low MW. An attempt is also made to establish correlations between morphology, microstructure and rheological properties of the synthesized PP reactor blends.

## 2. Experimental

### 2.1. Materials and Methods

The 2-Phenyl Indene, methyl aluminoxane (MAO, 10 wt % solution in toluene), *n*-BuLi, zirconium tetrachloride, and ethereal hydrochloric acid (1 N) were purchased from Aldrich and used as received. Triisobutyl aluminum (TIBA) and diethyl ether were obtained from Merck Chemical Co., Darmstadt, Germany. Polymerization grade propylene, *n*-hexane, *n*-heptane, and toluene were provided by Bandar Imam Petrochemical Co. (Tehran, Iran). The *rac*-Me_2_-Si(2-Me-4-Ph In)_2_ZrCl_2_ (I) was supplied by Molport, and (2-Ph In)_2_ZrCl_2_ (II), was synthesized according to a conventional technique described in the literature [38,39]. All solvents were refluxed over metallic sodium/benzophenone and distilled under nitrogen atmosphere prior to use. Propylene and nitrogen gas were dried by passing through columns containing a 3 Å molecular sieve. All reactions were performed under nitrogen atmosphere using the standard Schlenk technique and/or glovebox.

### 2.2. Catalyst Preparation

The catalyst precursors (I): *rac*-Me_2_-Si(2-Me-4-Ph In)_2_ZrCl_2_ and (II): (2-Ph In)_2_ZrCl_2_ were dissolved and homogenized in toluene. These solutions were used in propylene polymerization individually or in a mixed form with the predetermined Zr_(II)_/Zr_(I)_ molar ratios.

### 2.3. Polymerization

Polymerization reactions were performed in a 600 mL double walled stainless steel autoclave equipped with controlling systems for temperature, stirring speed, and reaction pressure. A certain amount of dried toluene (350 mL) was introduced into the reactor under N_2_ purge. A fixed amount of TIBA or MAO was injected into the reactor as a scavenger in all runs. Then, the reactor was pressurized with propylene gas up to 6 bar, and the solution was saturated by stirring at 500 rpm. After that, a specific volume of pre-activated catalyst with MAO (10 min pre-activation) was injected into the reactor via a high-pressure catalyst injecting system and the polymerization was started. The total pressure was kept constant during the reaction and polymerization time was 1 h. The reaction was terminated by stopping the propylene feed and depressurizing the reactor. Then, the polymerization mixture was quenched within the acidified methanol (2% HCl), washed with fresh methanol, filtered, dried and weighed.

### 2.4. Characterization

#### 2.4.1. Gel Permeation Chromatography (GPC)

MW and MWD of samples were measured by a high temperature size exclusion chromatography (SEC) technique on a PL-GPC 220 apparatus from Polymer Laboratories (Cambridge, UK) equipped with three columns. Samples with 1 mg/mL concentrations stabilized by 0.15 wt % butylated hydroxytoluene (BHT) were eluted with trichlorobenzene (TCB) at 145 °C and a flow rate of 1 mL/min.

#### 2.4.2. Xylene Solubility

The xylene solubility of polymers was determined by a conventional method described elsewhere [5].

#### 2.4.3. Rheological Properties

Dynamic rheometry in the melt state was carried out using a stress controlled rheometer (Anton Paar MCR301, Anton Paar GmbH, GRAZ, Austria) equipped with parallel plates geometry (diameter = 25 mm, gap = 1 mm). The samples were prepared as discs of 25 mm diameter and 2 mm thickness by compression molding. All measurements were done in a dry nitrogen atmosphere to suppress oxidative degradation. To achieve thermal equilibrium and structural relaxations, a waiting time after loading was applied before the measurements. Dynamic strain sweeps showed that rheological measurements performed at a strain of 5%, safely fall within the linear viscoelastic limit. Then, dynamic frequency sweeps were performed in the frequency range of 0.01–100 rad/s at a fixed temperature of 200 °C.

#### 2.4.4. Scanning Electron Microscopy (SEM)

Morphology of the samples was analyzed by scanning electron microscopy (SEM) (VEGA, TESCAN, Brno, Czech Republic).

#### 2.4.5. FTIR Analysis

Fourier transform infrared (FTIR) spectra were recorded on a Perkin–Elmer RXI spectrometer (Perkin–Elmer, Waltham, MA, USA) in the range of 1600–400 cm^−1^ with a resolution of 2 cm^−1^.

## 3. Results and Discussion

### 3.1. Polymerization

The polymerization results of catalysts (I) and (II) and their binary systems are tabulated in Table 1. It can be seen that the productivity of the binary catalyst increases with decreasing of Zr_(II)_/Zr_(I)_ molar ratio. This could be considered as a result of a higher portion of catalyst (I) and its higher activity as compared to catalyst (II).

### 3.2. Molecular Weight and Molecular Weight Distribution

**Figure 1:**
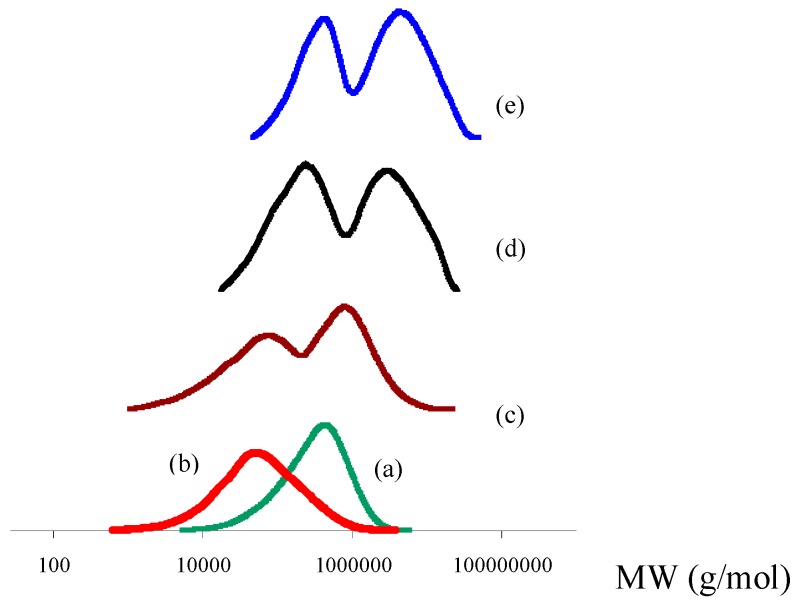
MWD Curves of the synthesized PP reactor blends by use of individual and binary catalysts: (**a**) PP-Cat I; (**b**) PP-Cat II; (**c**) PP-H Cat (25); (**d**) PP-H Cat (32); (**e**) PP-H Cat (40).

The MWD curves of polymers produced by the individual use of catalysts (I) and (II) and their binary systems are shown in Figure 1 and the quantitative values of average MWs and polydispersities are represented in Table 2. These curves show broad and also bimodal MWDs for the synthesized polymers by the binary catalysts. The appearance of two peaks in the MWD curves is indicative of the fact that the two catalysts are active during the polymerization in the case of the binary system. In addition, the average MWs are shifted to higher values in the polymer reactor blends. The increased MWs could be suggestive of decreased chain transfer reactions in two active catalytic sites and this feature is more remarkable for catalyst (I) (Table 2). Therefore, it can be concluded that catalysts were affected by each other since products with higher MW’s were obtained. When these two catalysts are mixed, the activity of catalyst (II) decreases (not reported here) and this decrease in activity can reduce the chain transfer and hence higher MWs products are obtained for the hybrid catalysts. For samples having a bimodal MWD, it is observed that the MWs at peaks (Table 2) are increased by increasing catalyst (II) content. The attained results indicate that the activity of binary catalysts, and as a consequence, MWs of the synthesized polymers, is highly sensitive to the molar ratios of catalysts. In these blends, the peak with high MW leads to products with considerable enhancement in mechanical properties, while the peak with low MW leads to an improvement in their processability. In other words, the increased tensile strength is accompanied by the improved processability.

### 3.3. FTIR Results

**Figure 2:**
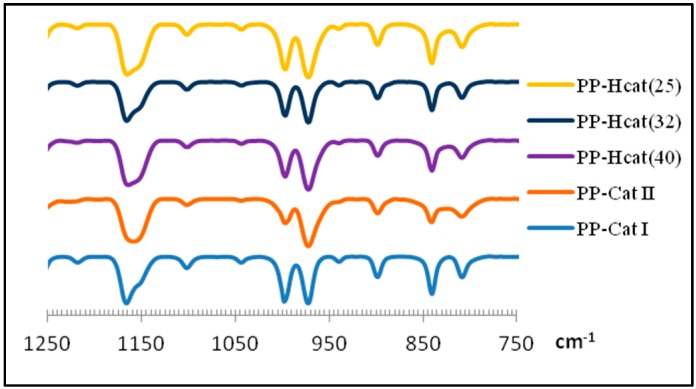
FTIR spectra of PP reactor blends synthesized with the binary and individual catalyst systems.

The FTIR spectra and corresponding data are reported in Figure 2 and Table 3, respectively. FTIR is one of the most common, simple and economical techniques for indirect measurement of the tacticity of PP. The 998 and 973 cm^−1^ absorption bands are characteristic bands assigned to helices chain conformation related to the isotactic segments that have different numbers of repeating units. The 998 and 973 cm^−1^ bands are assigned to segments with 11–12 and five repeating units, respectively. From the ratio of 998/973 absorption bands (A998/A973) known as the macrotacticity index and with the aid of the calibration curve, the PP tacticity can be evaluated [40,41,42,43].

The absorption band at 973 cm^−1^ is observed both in *i*-PP and *a*-PP with almost constant intensity. However, the intensity of the 998 cm^−1^ absorption band differs for these polymers. For this reason, the 973 cm^−1^ band is used as an internal reference while the A998/A973 ratio is used as the isotacticity index for PP. The intensity of the 998 cm^−1^ absorption band is proportional to the concentration of isotactic segments in PP chains. A decrease of an increase in the A998/A973 ratio is an indication of the reduction or enhancement of PP tacticity [41]. From the results presented in Table 3, it is obvious that the sample produced by catalyst (I) (PP-Cat I) is a PP with very high tacticity which shows FTIR bands typical of a commercial *i*-PP. Moreover, PP-Cat II is a PP with low tacticity content, exhibiting a band at 998 cm^−1^ with highly reduced intensity which is due to an inherent characteristic of catalyst (II) in producing polymers with large atactic blocks (segments).

FTIR spectra of PP-H Cat (25), PP-H Cat (32), and PP-H Cat (40) samples indicate that the A998/A973 ratio decreases with the increasing Zr_(II)_/Zr_(I)_ ratio. In the other words, due to the reduction of 998 cm^−1^ band intensity, the isotacticity of the synthesized products is reduced. This reduced isotacticity from PP-H Cat (25) to PP-H Cat (40) can be attributed to the increased content of catalyst (II) in the binary catalyst system and to its higher activity as compared to the catalyst (I) which leads to the generation of polymeric chains with higher atactic segments.

### 3.4. Linear Rheological Behavior

#### 3.4.1. Strain Sweep

**Figure 3:**
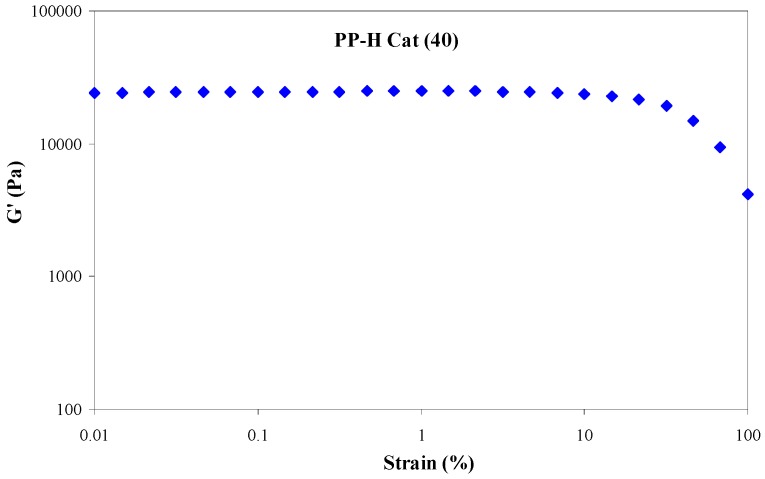
Storage modulus versus strain amplitude at a constant frequency of 10 rad/s and temperature of 200 °C.

Investigating the rheological behavior within the linear viscoelastic region is one of the most common methods for analyzing the structure of polymers. The variation of storage modulus in terms of strain amplitude for the synthesized PPs is shown in Figure 3 at a constant frequency of 10 rad/s within the strain range of 0.01%–100%. The attained results demonstrate that the linear viscoelastic region (LVR) occurs for the synthesized samples at strains lower than 20%. Therefore, in order to perform other dynamic sweeps and to avoid any non-linear responses, the strain amplitude was fixed at 5% to observe a more logical response at the low-frequency region.

The strain sweep test was reported for the PP-H Cat (40) sample since it has the highest MW among all samples. For this sample, it is shown that the strain of 5% safely falls within the linear viscoelastic region. The other samples which have lower MWs than the mentioned sample surely remain in the linear viscoelastic region at a strain of 5%.

#### 3.4.2. Frequency Sweep

**Figure 4:**
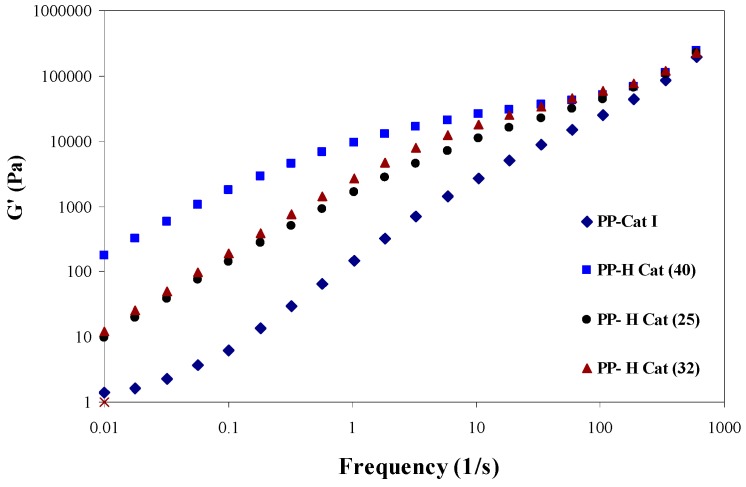
Storage modulus versus frequency for the PP reactor blends synthesized using binary metallocene catalysts at different molar ratios.

Figure 4 shows the storage modulus (*G*’) values, which are illustrated as a function of frequency within the linear viscoelastic region for the synthesized PPs using binary metallocene catalysts at different molar ratios of II/I (25, 32 and 40). According to this figure, the storage modulus values exhibit a rather similar variation trend for different samples within the whole frequency range. Moreover, all samples exhibit a modulus upturn at the low frequency region; similar to what is observed for the viscosity versus frequency curves presented subsequently [44,45,46]. Within the whole frequency range, the *G*’ values were found to be increased for all the synthesized PPs upon increasing the content of catalyst (II); albeit the majority of variations are observed within the low-frequency region. The observed plateau within the low-frequency region, which is associated with the solid-like behavior of the synthesized PPs induced by varying the composition of catalysts, is not observed for all the samples. As the molar ratio of II/I increases (from 25 to 40), the storage modulus within the low-frequency region exhibits a notable enhancement which could be attributed to the increased levels of MW and amorphous blocks in the synthesized PPs. These mentioned factors are themselves originated from the mixing of catalysts and their mutual effects on each other [47].

**Figure 5:**
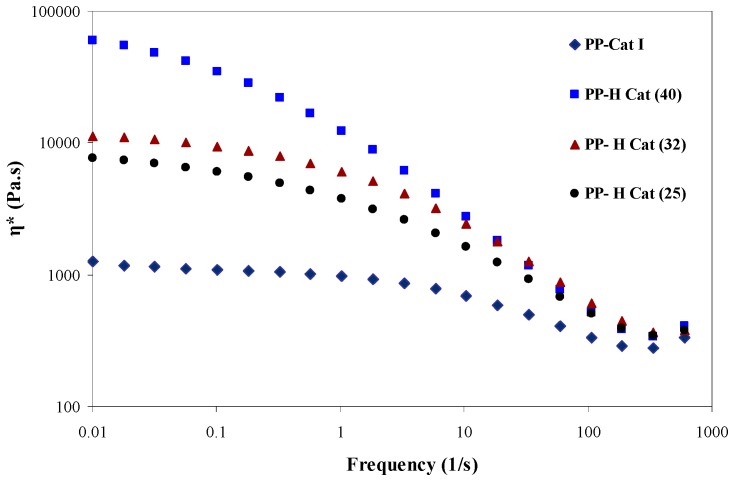
Complex viscosity versus frequency for the synthesized PP reactor blends.

**Figure 6:**
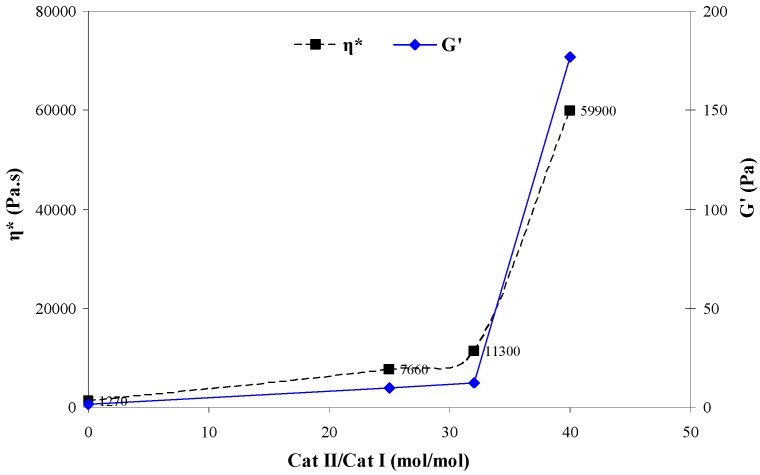
Storage modulus and complex viscosity as a function of molar ratio of the catalysts at a frequency of 0.01 rad/s.

The complex viscosity (η*) is depicted in Figure 5 as a function of frequency for the synthesized PPs using catalyst (I) and the binary catalysts. Based on the results, all the synthesized PP reactor blends exhibit the plateau region at low frequencies, indicating their Newtonian behavior. As the molar ratio of II/I is increased, their complex viscosities show remarkable increments within the low frequencies, and the shear-thinning behavior becomes more discernible for all the synthesized samples. The observed enhancement in complex viscosity for the samples could be ascribed to the increased MW together with the higher level of chains’ entanglements [48,49,50]. Figure 6 exhibits the complex viscosity and storage modulus versus catalyst molar ratios which shows that the complex viscosity of the synthesized blends increases almost 47-fold by increasing the molar ratio of II/I. Such enhancement in the storage modulus is also observed. Based on these results, one could conclude that upon the variation of the molar ratio of II/I, the microstructure of the growing chains was changed as compared with the produced polymer from the individual use of catalyst (I). This change has occurred in such a way that the amorphous blocks were formed along the polymer chains due to the presence of catalyst (II) and its activeness in the course of polymerization [47,51,52]. On the other hand, the formation of amorphous blocks along the polymer chains also leads to increased chains’ entanglements which are majorly responsible for the increased complex viscosity of the synthesized PP reactor blends [46,53,54].

**Figure 7:**
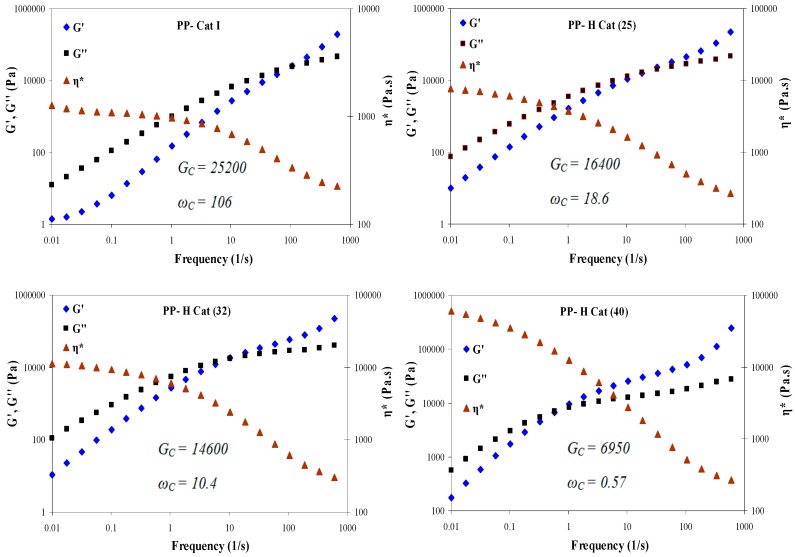
*G*’, *G*” and η* values in terms of frequency for the synthesized PP reactor blends with different molar ratios of cat (II)/cat (I).

The relaxation time spectra can be attained from the first cross-over frequency of *G*’ and *G*” curves ($\omega_{C_{1}}$). The cross-over point of *G*’ and *G*” curves is defined as the cross-over modulus (*G*_c_) which distinguishes the viscose behavior from the elastic behavior [45,54]. Based on the cross-over point of *G*’ and *G*” curves in terms of frequency, one could evaluate the viscoelastic behavior of the understudied blends. According to Figure 7, the first cross-over point ($G_{C_{1}}$), at very low frequencies, is originated from the increased elasticity of the polymer melt due to the chains’ entanglements [41]. From the observed cross-over point shift to the lower frequencies, it could be inferred that the melt elasticity is increased as a result of the increased level of intermolecular entanglements. It also can be stated that with increasing the melt elasticity, the relaxation time shifts towards longer times [50,51].

**Figure 8:**
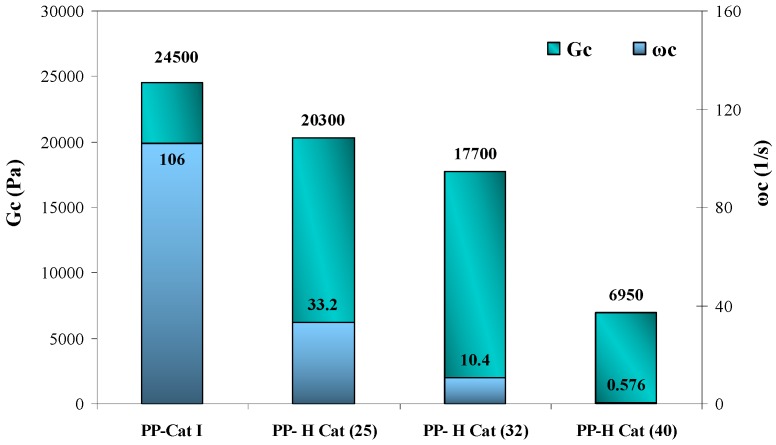
*G*_c_ and ω_C_ values for the synthesized PP reactor blends at different molar ratios of cat (II)/cat (I).

According to Figure 8, as the molar ratio of catalysts increases from 25 to 40, *G*_c_ and ω_c_ are changed from 24,500 and 106 to 6950 and 0.57, respectively. It can be concluded that with changing the molar ratio of II/I, the cross-over modulus and frequency are both decreased. The reason for the observed phenomenon could be ascribed to the higher elasticity of polymer melt due to the increased MW and also formation of amorphous blocks along the polymer chains [50].

#### 3.4.3. Effect of Increasing Molar Ratio of Cat (II)/Cat (I) on the Relaxation Time

**Figure 9:**
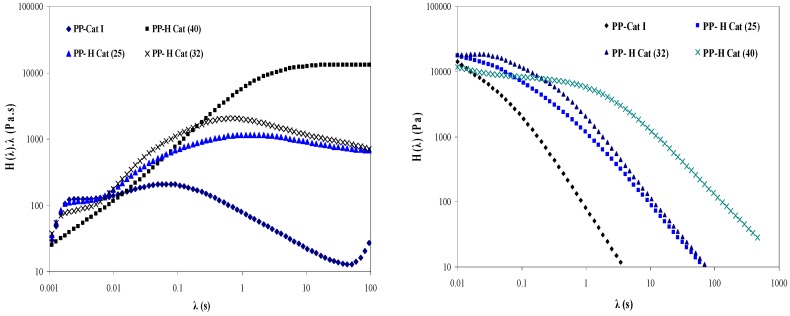
H(λ)λ and H(λ) for the PP reactor blends synthesized at different molar ratios of cat (II)/cat (I).

**Figure 10:**
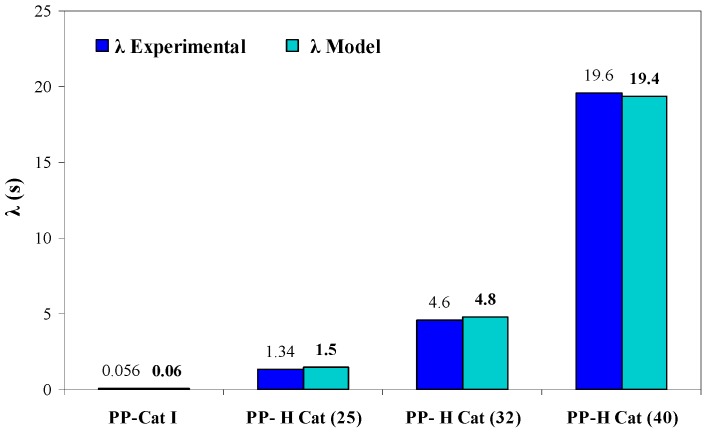
Relaxation time values for the PP reactor blends synthesized at different molar ratios of cat (II)/cat (I).

In Figure 9 and Figure 10, the relaxation spectra are illustrated in terms of λ within the linear viscoelastic region for the PP blends synthesized at different molar ratios of II/I. As the molar ratio of catalysts changes, all the samples exhibit an individual relaxation time peak which is shifted towards longer relaxation times with increasing the molar ratio of catalyst (II). Based on the obtained results, the PP-H Cat (40) sample exhibits the longest relaxation time (19.6 s) among the samples. The same trend is observed for the relaxation spectra of PP-H Cat (32) and PP-H Cat (25), and their relaxation times are 4.6 and 1.34 s, respectively.

According to Figure 9, it can be observed that the relaxation time and the heights of relaxation spectra H(λ)λ are increased for all the samples as the molar ratio of catalyst (II) is increased. The longer relaxation time can be attributed to the increased molecular weight of the samples. This indicates that increasing the molar ratio of catalyst (II) causes the elasticity to be increased due to the higher level of inter-chain entanglements leading to the longer relaxation time of chains after the shear flow is stopped. It can be concluded that increasing the molar ratio of II/I results in broadening the relaxation time spectrum and its consequent shift to the longer times. Therefore, the longer relaxation time, associated with the rigidity of the polymer chains, is due to the hindered motion of the chains as a result of chains’ entanglements, and accordingly, the sample with a molar ratio of 40 exhibits the longest relaxation time and the highest H(λ)λ peak height [45,46,50,54].

### 3.5. Correlation of Morphological and Rheological Properties

**Figure 11:**
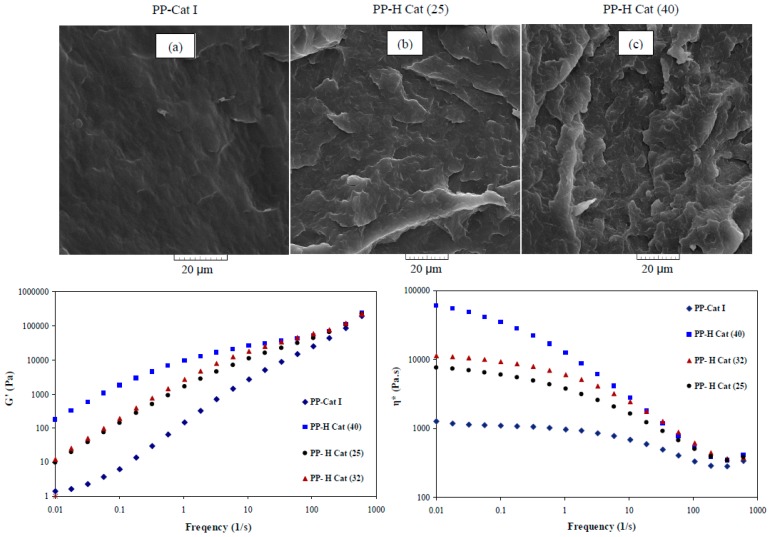
Relationship between the morphology and linear rheology of PP reactor blends synthesized at different cat (II)/cat (I) molar ratios.

Figure 11 reveals the existing relationship between the morphological features and rheological behavior of samples synthesized at different molar ratios of II/I. The morphological features of the synthesized PPs via Cat (I) and the binary metallocene catalysts (25 and 40) are shown at the top of Figure 10. As can be seen, with increasing the molar ratio of catalyst (II) to catalyst (I), the morphology becomes more heterogeneous. Such morphological inhomogeneity is originated from the effect of increasing the content of catalyst (II) on the growing chains and also the effect of irregular atactic blocks formed during the course of polymerization as compared to the isotactic PP. In other words, the heterogeneity in the microstructure can be attributed to the formation of amorphous blocks along the polymer chains due to the activity of the catalyst (II) within the binary catalyst systems and also the increased MW of the samples which itself might be the major reason for the higher complex viscosity and storage modulus within the low-frequency region and also the higher ductility of polymer chains.

According to Figure 11, the observed changes in the morphological features, induced from increasing the II/I molar ratio, are further confirmed by the obtained dynamic rheological data. Therefore, it can be concluded that there exists a promising relationship between the linear rheological data and morphological features such that the chains’ elasticity is notably increased as the II/I molar ratio increases, especially at the low-frequency region [46,50,54].

## 4. Conclusions

The following conclusion can be drawn from the obtained results in the current study:

1. The MWD curves of synthesized products by the individual use of catalysts (I) and (II) and their binary systems show that MWD was broadened and became bimodal for products obtained from the catalysts’ mixtures. In addition, the average MWs were shifted to higher values in PP reactor blends. Actually, the appearance of two peaks in the MWD curves is indicative of the fact that two catalysts are active during the polymerization in the case of binary systems. In these blends, the peak at higher MW leads to a product with remarkable enhancement in mechanical properties, while the peak at lower MW leads to a product with improved processability. In other words, the improved tensile strength of the products is accompanied by their improved processability.
2. The complex viscosity of the synthesized blends shows a remarkable 47-fold enhancement as the molar ratio of II/I is increased. It can be concluded that upon the changes in the molar ratio of catalyst (II), the structure of the growing chains is changed such that the amorphous blocks along the polymer chains are formed due to the presence of catalyst (II) and its activeness in the course of polymerization. The formation of those amorphous blocks along the polymer chains increases the chains’ entanglements and thus enhances the complex viscosity of the synthesized PP reactor blends.
3. As the molar ratio of II/I changes, the cross-over modulus and frequency are reduced due to the increased elasticity of the polymer melt as a result of the existence of amorphous blocks along the polymer chains.
4. With increasing the molar ratio of II/I, all the peaks exhibited an individual relaxation peak which tends towards longer times as the molar ratio of catalyst (II) is increased. As a result, PP-H Cat (40) exhibits the longest relaxation time among the samples.
5. The changes in morphological features of obtained products induced by the increased molar ratio of II/I in the synthesized blends was precisely demonstrated via SEM images and also dynamic rheological data. Therefore, it can be concluded that there exists a robust relationship between the obtained results of linear rheology and morphological characteristics such that with increasing the molar ratio of the catalyst (II) within the binary system, the chains’ elasticity was significantly increased. It was also found that the amount of the mentioned enhancement within the low-frequency region is dependent on the formation of amorphous blocks along the PP chains.

## Figures and Tables

**Table 1:** Polymerization results of catalysts (I) and (II) and their binary systems.

Run	Sample rode	Zr_(II)_/Zr_(I)_ (Molar ratio)	Zr_(t)_ × 10^4^ (mmol)	Al/Zr_(t)_	Polymer wt (g)	Productivity (kg PP/mmol Zr_(t)_·h)
1	PP-Cat I	(I) only	3.5	1100	10.0	28.8
2	PP-Cat II	(II) only	75	1100	6.0	0.80
3	PP-H Cat (25)	25	45.6	1100	54.3	11.9
4	PP-H Cat (32)	32	57.8	1100	66.5	11.5
5	PP-H Cat (40)	40	71.8	1100	30.9	4.3

Polymerization condition: Toluene = 350 mL, *P* = 6 bar, *T* = 40 °C, *t* = 60 min, methyl aluminoxane (as Scavenger) = 1 mL, rpm = 500, pre-activation time: 10 min.

**Table 2:** MW and MWD of the synthesized PP reactor blends.

Run	Sample code	Zr_(II)_/Zr_(I)_	MW (g/mol)	PDI ^a^	Peak max		Xylene solubility (wt %)
					Low MW (g/mol)	High MW (g/mol)	
1	PP-Cat I	(I) only	469,000	2.8	383,000 ^b^		2.6
2	PP-Cat II	(II) only	115,000	4.5	43,000 ^b^		84
3	PP-H Cat (25)	25	1,340,000	b.m ^c^	66,000	704,000	23
4	PP-H Cat (32)	32	2,850,000	b.m	201,000	2,410,000	28
5	PP-H Cat (40)	40	4,420,000	b.m	366,000	4,120,000	35

^a^ Polydispersity Index, ^b^ Unimodal, ^c^ Bimodal.

**Table 3:** FTIR results of PP reactor blends synthesized with the binary and individual catalyst systems.

Sample code	Zr_(II)_/Zr_(I)_	A998 (Peak height)	A973 (Peak height)	A998/A973	Isotacticity ^1^	Isotacticity ^2^
PP-Cat I	(I) only	64.7	75.2	0.86	96.8	97.4
PP-Cat II	(II) only	7.8	77.9	0.10	20	16
PP-H Cat (25)	25	49.1	74.4	0.66	76.2	77
PP-H Cat (32)	32	43.5	76.3	0.57	70.1	72
PP-H Cat (40)	40	40.4	77.6	0.52	63.3	65

^1^ Calculated from the calibration curve reported in [41]; ^2^ From xylene solubility.
